# Comparison of the Effects of Subconjunctival Injections of Bevacizumab and Interferon Alpha-2a on Corneal Angiogenesis in a Rat Model

**DOI:** 10.3390/medicina54020016

**Published:** 2018-04-16

**Authors:** Sinan Bilgin

**Affiliations:** Department of Ophthalmology, Sifa University, Izmir 35100, Turkey; drsinanbilginsule@gmail.com; Tel.: +90-505-432-1725; Fax: +90-232-444-2002

**Keywords:** corneal neovascularization rate, corneal neovascular sprout length, bevacizumab, interferon alpha-2a

## Abstract

*Background and objective:* Corneal neovascularization (CNV) is a vision-threatening condition arising from various corneal diseases. The aim of this study is to compare the effectiveness of bevacizumab and interferon alpha-2a (IFNα-2a) treatment on corneal neovascularization. *Materials and Methods:* Twenty-four Wistar albino rats were used in this study. After cauterization of the cornea with a silver nitrate applicator stick, the control group received 0.1 mL saline solution, the second group received 0.1 mL IFNα-2a (IFNα-2a, 6 million international units [MIU]/0.5 mL), and the third group received 2.5 mg bevacizumab by subconjunctival injection. An additional injection was administered to each group on the fourth day. After one week, the corneal neovascularization rate and the longest neovascular sprout length were determined. *Results:* The neovascularization rate (saline 0.65 ± 0.05; IFNα-2a 0.62 ± 0.07; bevacizumab 0.42 ± 0.11) with bevacizumab was significantly lower, more than those with IFNα-2a and saline (*p* < 0.001 and *p* < 0.001). The longest neovascular sprout length (saline, 4.00 ± 0.6 mm; IFNα-2a, 3.63 ± 0.52 mm; bevacizumab, 2.81 ± 0.65 mm) with bevacizumab was significantly shorter than those with saline and IFNα-2a (*p* = 0.001 and *p* = 0.012). *Conclusions:* Subconjunctival IFNα-2a has limited efficacy in the treatment of corneal neovascularization.

## 1. Introduction

Corneal neovascularization (CNV) is a vision-threatening situation arising from a variety of traumatic, infectious, inflammatory, and degenerative ocular surface disorders. It is hard to treat CNV for eye doctors [1]. 

Various mediators including vascular endotehelial growth factor (VEGF), matrix metalloproteases, and cytokines have been implicated in the stimulation of new vessel formation [2,3,4]. A variety of pharmacotherapies including anti-VEGF drugs, triamcinolone acetonide, tetracycline derivatives, and multi-tyrosine kinase inhibitor (TKI) have been studied as potential CNV inhibitors [5,6,7,8].

Recently, to diminish corneal neovascularization, investigators have increasingly used VEGF inhibitors. The effectiveness of bevacizumab treatment on corneal neovascularization has been demonstrated by two studies [8,9]. Interferons (IFNs) are cytokines and released by host cells. They have antiproliferative and immunomodulatory features. IFNs have been shown to inhibit VEGF and other cytokines including interleukins and tumor necrosis factor alpha (TNF-α), while increasing the impermeability of the retinal vascular structure [10,11]. Owing to the effects of anti-VEGF and the anti-proliferative, interferon alpha-2a (IFNα-2a) has been used in patients with ocular neovascular diseases [12,13]. There have been few reports about the use of IFNs in the anterior segment of the eye.

Ross et al., using IFN-α immunotherapy, obtained sufficient outcomes in the treatment of conjunctival, mucosa-associated lymphoid tissue lymphoma [14]. Successful antitumor activity of IFN-α has been demonstrated in these studies, and there have been some case reports demonstrating the therapeutic effect of IFN-α on macular edema in diabetic patients and wet type macular degeneration [12,13]. However, to the best of our knowledge, there have been no studies that have examined the efficacy of IFN-α in CNV. The aim of this study is to compare the effectiveness of bevacizumab and interferon alpha-2a treatment on corneal neovascularization in experimental corneal injury in a rats.

## 2. Materials and Methods

We used 24 Wistar albino rats without corneal lesions. All rats were separated into three groups, and every group had eight rats. Animals were housed in plastic cages in a temperature-controlled place, and all cages had a 12/12 h light-dark period (07:00–19:00 light). They were treated in accordance with the Association for Research in Vision and Ophthalmology’s Statement for the Use of Animals in Ophthalmic and Vision Research. The Institutional Animal Care and Use Committee certified the study design (2014-032).

The rats were anesthetized using a combination of ketamine hydrochloride (50 mg/kg) and xylazine (5 mg/kg). After topical anesthetize with 0.5% proparacaine hydrochloride, corneal wound was performed in accordance with a model defined by Kwon et al. only in the right eye of each subject [15]. A standard cornel insult was produced by touching the central cornea with an applicator stick covered with 25% potassium nitrate and 75% silver nitrate for 10 s (with a diameter of 2 mm) using an operating microscope. The fornix and cornea were then washed with an isotonic saline to enhance the reproducibility of the wounds. All rats were cauterized by the same researcher. Immediately following cauterization, the control group received a 0.1 mL isotonic saline injection, the second group received a 0.1 mL IFNα-2a injection (6 million international units (MIU)/0.5 mL), and the third group received 2.5 mg of bevacizumab by subconjunctival injection. Second injections were performed in all groups on the fourth day. All subjects were examined by biomicroscopy (Topcon, DC-3, Tokyo, Japan) on only three separate occasions (1st, 4th, and 8th day), due to the difficulties and risks associated with anesthesia.

No obvious neovascularization was observed by the 4th day; therefore, anterior segment photos were only taken on the eighth day using a Topcon digital camera (BG-4 model) mounted on a biomicroscopy unit to determine the extent of corneal neovascularization. All images were taken at 12X magnification, from the same distance (10 cm) and under the same lighting by the same observer. The images were assessed by a single independent observer who was blinded to the treatment group. The images were analyzed using the software program Image J 1.46 (created by Wayne Rasband at the Research Services Branch, National Institute of Mental Health, Bethesda, MD, USA).

**Figure 1:**
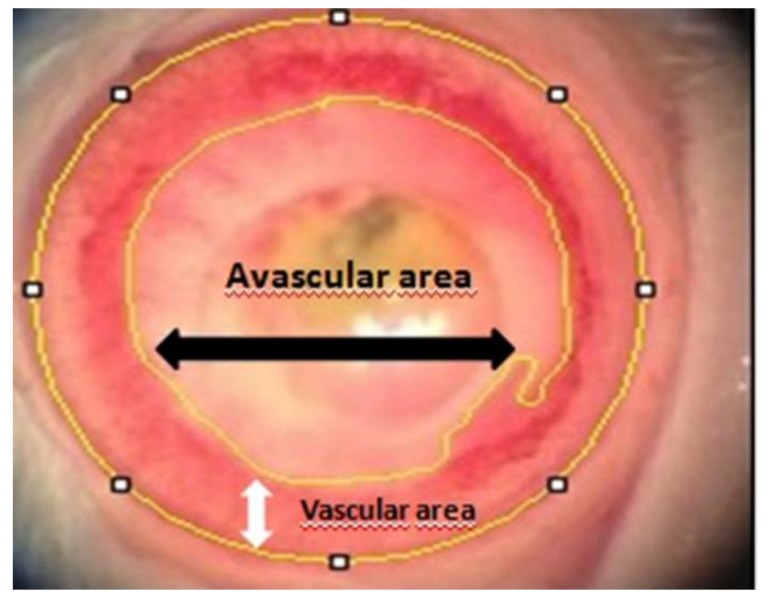
Vascular and avascular corneal area are outlined with reference to the corneal limbus. Black arrow shows avascular area, and white arrow shows vascular area.

The total corneal area and avascular corneal area were outlined with reference to the corneal limbus (Figure 1). The number of pixels in the avascular area was subtracted from the number of pixels in the total corneal area, and the resulting value was divided by the number of pixels in the total corneal area. The resultant value was accepted as the neovascularization rate [16]. The longest neovascular sprout length in corneas was assessed. Using a lethal dose of pentobarbital, all the subjects were sacrificed at the end of the study.

### Statistical Analysis

The SPSS 24.0 (IBM Corporation, Armonk, NY, USA) program was used to analyze the variables. The conformity of the data to normal distribution was evaluated by the Shapiro-Wilk test, while the variance homogeneity was evaluated by the Levene test. The One-Way Anova (Robust Tests: Brown-Forsythe) test was used in the comparison of the groups with each other according to the neovascularization rate and the longest neovascular sprout length, the quantitative data, while the Fisher’s Least Significant Difference test was used for the Post Hoc analyses. The quantitative variables were shown as mean ± s.d. (standard deviation), and the categorical variables were shown as *n* (%) in the tables. The variables were examined at 95% confidence level, and *p* < 0.05 was accepted as significant.

## 3. Results

Only data from day eight were used in the statistical analysis, because there was insufficient neovascularization on the fourth day to accurately measure the percentage of neovascularization of the cornea. The neovascularization rate was 0.65 ± 0.05 in the saline group, 0.62 ± 0.07 in the IFNα-2a group, and 0.42 ± 0.11 in the bevacizumab group. There were significant differences in the neovascularization rate between the three treatment groups (*p* < 0.001) (Table 1).

**Figure 2:**
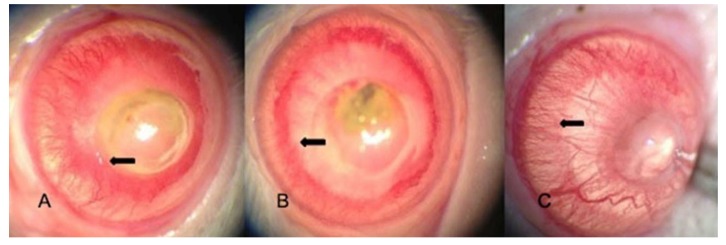
Representative photographs of the cornea on the eighth day of the treated groups. (**A**) Saline, (**B**) INFα-2a, (**C**) Bevacizumab. Bevacizumabe group has more clear cornea, less corneal inflammation, and less neovascularization (compare arrows) according to saline and IFNα-2a groups.

The neovascularization rate was significantly lower in the bevacizumab group compared with the IFNα-2a and saline groups (*p* < 0.001, *p* < 0.001). The difference in the neovascularization rate between the IFNα-2a group and saline group was not significant (*p* = 0.422). Figure 2 shows the corneal photographs from animals with neovascularization. The length of the longest neovascular vessel was 4.00 ± 0.6 mm in the saline group, 3.63 ± 0.52 mm in IFNα-2a group, and 2.81 ± 0.65 mm in the bevacizumab group (Table 1).

There were significant differences in the longest neovascular sprout length among the three treatment groups (*p* = 0.002). The longest neovascular sprout length was significantly shorter in the bevacizumab group when compared with the saline and IFNα-2a groups (*p* = 0.001, *p* = 0.012 respectively). Although the longest neovascular sprout length in the IFNα-2a group was shorter than that in the saline group, this difference was not statistically significant (*p* = 0.219) (Table 1). 

## 4. Discussion

The preservation of corneal clarity depends on the maintenance of a precise balance between angiogenic and anti-angiogenic factors. Any interruption to this balance can result in invasion of vessels into the cornea. VEGF is a vasoproliferative factor. When VEGF is activated, corneal neovascularization launches; however, inhibition of VEGF decelerates neovascularization [2,3]. In some studies, including animal models of corneal NV, an increase in the amount of VEGF has been demonstrated [17,18]. Studies have shown that corneal sprouts with NV have higher accumulation of VEGF, and their receptors accord to normal corneas, regardless of the reason of neovascularization [19]. 

Various therapeutic agents including anti-VEGF, steroids, doxycycline, tigecycline, minocycline, pazopanib, and indomethacin have been demonstrated to be efficacious at blocking corneal neovascularization pathways [5,6,7,8,20,21]. Bevacizumab has been used at different concentrations and over different treatment periods, either topically or subconjunctivally, in the treatment of corneal NV and has been shown to be efficient in decreasing neovascularization. It has been reported that bevacizumab is effective, at both 1.25 mg and 2.5 mg, for reducing corneal NV [8,19,22]. One potential antiangiogenic treatment is IFN therapy. It acts as an antiangiogenic agent by inhibiting the migration and proliferation of endothelial cells. Due to these features, interferons have been used to treat non-infectious uveitic chronic macular edema and conjunctival lymphoma [14,23]. It has been reported that systemic IFNα-2a treatment is effective in the treatment of Behçet disease-associated uveitis. Virtually all the patients were found to show complete or partial remission [23,24]. However, visual acuity was not improved in most patients [24]. Cellini et al. showed that the use of subtenon injections of IFNα-2a is effective at treating diabetic macular edema that is refractory to laser grid treatment and intravitreal injections of triamcinolone, with no adverse events recorded [12]. Systemic IFN-α has also been shown to inhibit iris neovascularization in an animal model [25]. Although our results were better for the IFNα-2a group, the differences between IFNα-2a and the control group were not significant for the neovascularization rate and the longest neovascular sprout length. In the studies mentioned above, IFNα-2a was administered three times a week or daily, while we employed a less frequent dosage regime. If the half-lives of IFNα-2a and bevacizumab are taken into account, it might be inferred that the less frequent administration of IFNα-2a might be one of the main reasons for the lack of significance. In the present study, in agreement with previous studies, bevacizumab was found to be very effective at treating CNV. The size of the neovascularization area was significantly smaller in the bevacizumab group compared with the IFNα-2a and control groups. The longest neovascular sprout length was also significantly smaller in the bevacizumab group when compared with the control and IFNα-2a groups. 

This study had some limitations including a relatively small sample size, and the choice of an infrequent high-dose INFα-2a therapeutic regimen. It has been shown that more frequent injections of low-dose IFNα have a strong anti-proliferative effect on cells; however, due to the difficulties in providing anesthesia, we had to use the treatment regimen reported by Cellini et al. Another factor is the difference in elimination half-life; the elimination half-life of standard IFNα is 3.8 to 7.3 h after IV infusion, while it is 20 days for bevacizumab [12]. Because of this, IFNα injections must be administered at frequent intervals [26,27]. The effectiveness of various dosages of IFNα-2a and bevacizumab in the treatment of CNV have not been evaluated. Also, in this study, a humanized anti-VEGF antibody (bevacizumab) has been used that connects defectively to murine VEGF-A. It has been shown that this antibody binds rat VEGF-A with less specificity and dissociates more easily than human VEGF-A [28]. Although CNV can be clearly observed, it would have been preferable to stain the corneas immunohistochemically with anti-CD31 antibody using whole mounts in order to get more precise results [29].

## 5. Conclusions

Although there have been many studies demonstrating the effectiveness of the bevacizumab treatment on corneal neovascularization, within our knowledge, this is the first study about the efficacy of IFNα-2a in the treatment of CNV. On the basis of our results, we feel that IFNα-2a has a very limited efficacy in the treatment of CNV. However, it should be emphasized that this study has some considerable limitations, and further studies are needed to better clarify the act of IFNα-2a in corneal neovascularization.

## Figures and Tables

**Table 1:** Evaluation of the groups according to the neovascularization rate and the longest neovascular sprout length.

Group			Neovascularization Rate	Longest Neovascular Sprout Length (mm)
	*n* (%)		Mean ± SD	Mean ± SD
Saline	8 (33.33)	=A	0.65 ± 0.05	4.00 ± 0.60
INFalpha 2a	8 (33.33)	=B	0.62 ± 0.07	3.63 ± 0.52
Bevacizumab	8 (33.33)	=C	0.42 ± 0.11	2.81 ± 0.65
*p* Value			<0.001 *	0.002 *
Pairwise comparation	A → B		0.422	0.219
	A → C		<0.001 *	0.001 *
	B → C		<0.001 *	0.012 *

One-Way ANOVA (Robust Tests: Brown-Forsythe); Post Hoc Test: Fisher’s Least Significant Difference; Values are expressed as mean ± SD; * statistically significant differences.
